# Genotypic tropism testing in proviral DNA to guide maraviroc initiation in aviremic subjects: 48-week analysis of the PROTEST study

**DOI:** 10.7448/IAS.17.4.19520

**Published:** 2014-11-02

**Authors:** Federico Garcia, Eva Poveda, Maria Jesús Pérez-Elías, José Hernández Quero, Maria Àngels Ribas, Onofre J Martínez-Madrid, Juan Flores, Manel Crespo, Félix Gutiérrez, Miguel García-Deltoro, Arkaitz Imaz, Antonio Ocampo, Arturo Artero, Francisco Blanco, Enrique Bernal, Juan Pasquau, Carlos Mínguez-Gallego, Núria Pérez, Aintzane Aiestarán, Roger Paredes

**Affiliations:** 1 Hospital Universitario San Cecilio, Granada, Spain; 2 INIBIC-Complexo Hospitalario Universitario de A Coruña, A Coruña, Spain; 3 Hospital Ramón y Cajal, Madrid, Spain; 4 Hospital Son Espases, Palma de Mallorca, Spain; 5 Hospital General Universitario Santa Lucía, Cartagena, Spain; 6 Hospital Arnau de Vilanova, Valencia, Spain; 7 Hospital Universitari Vall d'Hebron, Barcelona, Spain; 8 Hospital Universitario de Elche, Elche, Spain; 9 Hospital General Universitario de Valencia, Valencia, Spain; 10 Hospital de Bellvitge, Barcelona, Spain; 11 Hospital Xeral de Vigo, Vigo, Spain; 12 Hospital Universitario Dr. Peset, Valencia, Spain; 13 Hospital Carlos III, Madrid, Spain; 14 Hospital Reina Sofía, Murcia, Spain; 15 Hospital Virgen de la Nieves, Granada, Spain; 16 Hospital General de Castelló, Castelló, Spain; 17 Hospital Universitari Germans Trias i Pujol, Badalona, Spain; 18 irsiCaixa AIDS Research Institute, Badalona, Spain

## Abstract

**Introduction:**

In a previous interim 24-week virological safety analysis of the PROTEST study [1], initiation of Maraviroc (MVC) plus 2 nucleoside reverse-transcriptase inhibitors (NRTIs) in aviremic subjects based on genotypic tropism testing of proviral HIV-1 DNA was associated with low rates of virological failure. Here we present the final 48-week analysis of the study.

**Methods:**

PROTEST was a phase 4, prospective, single-arm clinical trial (ID: NCT01378910) carried on in 24 HIV care centres in Spain. Maraviroc-naïve HIV-1-positive adults with HIV-1 RNA (VL) <50 c/mL on stable ART during the previous 6 months, requiring an ART change due to toxicity, with no antiretroviral resistance to the ART started, and R5 HIV by proviral DNA genotypic tropism testing (defined as a G2P FPR >10% in a singleton), initiated MVC with 2 NRTIs and were followed for 48 weeks. Virological failure was defined as two consecutive VL>50 c/mL. Recent adherence was calculated as: (# pills taken/# pills prescribed during the previous week)*100.

**Results:**

Tropism results were available from 141/175 (80.6%) subjects screened: 87/141 (60%) were R5 and 74/87 (85%) were finally included in the study. Their median age was 48 years, 16% were women, 31% were MSM, 36% had CDC category C at study entry, 62% were HCV+ and 10% were HBV+. Median CD4+ counts were 616 cells/mm^3^ at screening, and median nadir CD4+ counts were 143 cells/mm^3^. Previous ART included PIs in 46 (62%) subjects, NNRTIs in 27 (36%) and integrase inhibitors (INIs) in 1 (2%). The main reasons for treatment change were dyslipidemia (42%), gastrointestinal symptoms (22%), and liver toxicity (15%). MVC was given alongside TDF/FTC in 40 (54%) subjects, ABC/3TC in 30 (40%), AZT/3TC in 2 (3%) and ABC/TDF in 2 (3%). Sixty-two (84%) subjects maintained VL<50 c/mL through week 48, whereas 12 (16%) discontinued treatment: two (3%) withdrew informed consent, one (1%) had a R5→X4 shift in HIV tropism between the screening and baseline visits, one (1%) was lost to follow-up, one (1%) developed an ART-related adverse event (rash), two (3%) died due to non-study-related causes (1 myocardial infarction at week 0 and 1 lung cancer at week 36), and five (7%) developed protocol-defined virological failure, although two of them regained VL<50 c/mL with the same MVC regimen (Table 1).

**Conclusions:**

Initiation of MVC plus 2 NRTIs in aviremic subjects based on genotypic tropism testing of proviral HIV-1 DNA is associated with low rates of virological failure up to one year.

**Table 1:** 

Subject	ART	Week of VF	HIV-1 VL at VF (c/mL)	Plasma tropism at VF (FPR,%)	Recent adherence at VF (%)	Resistance mutations at VF (IAS-USA 2013)	ART after VF	Regained VL <50 c/mL
1	MVC+TDF/FTC	4	300	X4 (0.1)	100	NA	DRV/r+ETV	Yes
2	MVC+TDF/FTC	12	14,102	X4 (1.3)	100	RT: 41L, 67N, 184V, 215Y PR: 36I, 63P	TDF/FTC+ETV	Yes
3	MVC+ABC/3TC	12	67	R5 (86.8)	100	RT: 90I, 184I PR: 64V	TDF+DRV/r+EFV	Yes
4	MVC+TDF/FTC	12	59	NA	100	NA	Continued with MVC+TDF/FTC	Yes
5	MVC+TDF/FTC	36	90	R5 (79.7)	100	NA	Continued with MVC+TDF/FTC	Yes

## References

[1] Federico Garcìa, Eva Poveda, Maria Ångels Ribas, María Jesús Pérez-Elías, Onofre J. Martínez-Madrid, Jordi Navarro (2014). Genotypic tropism testing of proviral DNA to guide maraviroc initiation in aviremic subjects. 21st Conference on Retroviruses and Opportunistic Infections 2014.

